# Association between genetic variants in the *XPG* gene and gastric cancer risk in a Southern Chinese population

**DOI:** 10.18632/aging.101119

**Published:** 2016-12-06

**Authors:** Rui-Xi Hua, Zhen-Jian Zhuo, Jinhong Zhu, Dan-Hua Jiang, Wen-Qiong Xue, Shao-Dan Zhang, Jiang-Bo Zhang, Xi-Zhao Li, Pei-Fen Zhang, Wei-Hua Jia, Guo-Ping Shen, Jing He

**Affiliations:** ^1^ Sun Yat-sen University Cancer Center, State Key Laboratory of Oncology in South China, Department of Experimental Research, Collaborative Innovation Center for Cancer Medicine, Guangzhou 510060, Guangdong, China; ^2^ Department of Pediatric Surgery, Guangzhou Institute of Pediatrics, Guangzhou Women and Children's Medical Center, Guangzhou Medical University, Guangzhou 510623, Guangdong, China; ^3^ Department of Oncology, The First Affiliated Hospital of Sun Yat-sen University, Guangzhou 510080, Guangdong, China; ^4^ School of Chinese Medicine, Faculty of Medicine, The Chinese University of Hong Kong, Hong Kong 999077, China; ^5^ Molecular Epidemiology Laboratory and Department of Laboratory Medicine, Harbin Medical University Cancer Hospital, Harbin 150040, Heilongjiang, China; ^6^ Department of Medical Genetics, Zhongshan School of Medicine, Sun Yat-sen University, Guangzhou 510080, Guangdong, China; ^7^ Department of Radiation Oncology, The First Affiliated Hospital of Sun Yat-sen University, Guangzhou 510080, Guangdong, China

**Keywords:** *XPG*, gastric cancer, polymorphism, genetic susceptibility

## Abstract

Xeroderma pigmentosum group G (XPG) recognizes and excises DNA damage on the 3′ side during the DNA repair process. Previous studies indicated that *XPG* gene polymorphisms may associate with gastric cancer susceptibility, but results were inconsistent. We evaluated the association of five potentially functional *XPG* polymorphisms (rs2094258 C>T, rs751402 C>T, rs2296147 T>C, rs1047768 T>C, and rs873601 G>A) with gastric cancer susceptibility in 1142 gastric cancer cases and 1173 controls. Odds ratios (ORs) and 95% confidence intervals (CIs) were calculated using logistic regression models. Overall, no significant association was detected between any of selected polymorphism and gastric cancer risk. However, we found that individuals carrying 3-4 risk genotypes were at significantly higher risk of gastric cancer than those with 0-2 risk genotypes (OR=1.32, 95% CI=1.04-1.68, *P*=0.021). The stratification analysis revealed that the cumulative effect of risk genotypes (3-4 vs. 0-2) on gastric cancer were more prominent among subgroups older than 58 years and men. In conclusion, our results indicated that none of the selected *XPG* polymorphism could significantly alter gastric cancer susceptibility alone. These polymorphisms might collectively confer increased gastric cancer susceptibility. These findings would be strengthened by larger prospective multicenter studies involving different ethnic populations.

## INTRODUCTION

Gastric cancer is one of the most common cancers with high mortality, ranking as the fifth most common and the third deadliest cancer in the world [1]. Decreasing trends in gastric cancer incidence and mortality have been reported in most industrialized countries, whereas it is still prevalent in developing countries, predominantly in China [2]. Despite remarkable progress achieved in multimodal therapy strategies, the survival of gastric cancer remains poor with overall 5-year survival rates hovering around 25% [3]. *Helicobacter pylori* (*H. pylori*) infection is a well-established risk factor for gastric cancer. However, some countries with a high *H. pylori* infection rate have disproportionately low gastric cancer incidence or mortality [4-6]. These observations suggested that rather than any single factor alone, the development of gastric cancer stem from a combination of multiple factors, such as *H. pylori* infection, nutritional deficiencies, a high salt or a low fiber diet, smoking, alcohol consumption, high body mass index [7, 8], and genetic predisposition [9].

DNA repair system is responsible for maintaining the stability and integrity of human genomic DNA [10], and DNA repair genes may serve as potential biomarkers for cancer predication and prognosis [11]. Nucleotide excision repair (NER), one of the highly evolutionarily conserved pathway, can monitor and repair a variety of DNA damages [12, 13]. Failure to repair DNA damages may lead to a number of human diseases including xeroderma pigmentosum (XP) [14]. X*eroderma pigmentosum group G* (*XPG*) gene is one of eight key genes [*XPA* to *XPG*, and excision repair cross complementing group-1 (*ERCC1*)] in the NER pathway [15]. XPG can recognize and cut DNA lesion on the 3′ side to ensure the proper repair of damaged DNA [16, 17]. XPG also serves as a nonenzymatic scaffolding for subsequent 5′ incision by the XPF/ERCC1 heterodimer during the NER process [18].

Thus far, a number of studies have reported the relationship between single nucleotide polymorphisms (SNPs) in the *XPG* gene and cancer risk, including lung cancer [19, 20], gastric cancer [21-24], esophageal squamous cell carcinoma [25], colorectal cancer [26-28], and neuroblastoma [29]. However, only a few papers with small sample sizes are available regarding the role of *XPG* gene SNPs in gastric cancer carcinogenesis, and conclusions remain conflicting [21-24]. Therefore, we performed this study to precisely determine the association between five potentially functional SNPs (rs2094258 C>T, rs751402 C>T, rs2296147 T>C, rs1047768 T>C and rs873601G>A) in the *XPG* gene and gastric cancer susceptibility with a total of 1142 patients and 1173 cancer-free controls in a Southern Chinese population.

## RESULTS

### Population characteristics

The final analysis consisted of 1142 cases and 1173 healthy controls (Supplemental Table S1). There were 65.59% and 67.26% men in cases and controls (*P=*0.393), respectively. However, regarding age, smoking status, drinking status, and pack-years, there existed significant difference (*P<*0.0001) between the cases and controls. Thus, we further adjusted for these variables in the multivariate analyses. Of the gastric cancer patients, 240 (21.02%) cases were diagnosed with gastric cardia adenocarcinoma, while 902 (78.98%) cases were with non-gastric cardia adenocarcinoma. In term of stage, 140 (12.26%), 329 (28.81%), 456 (39.93%), and 217 (19.00%) cases were classified as TNM stage I, II, III, and IV, respectively, according to the 7^th^ Edition of the American Joint Committee on Cancer (AJCC) [30].

### Associations between *XPG* gene polymorphisms and gastric cancer risk

The genotype frequencies of cases and controls for the five *XPG* SNPs and their associations with gastric cancer risk were summarized in Table 1. Observed genotype frequency distributions of all SNPs among the control subjects were in agreement with Hardy-Weinberg equilibrium (HWE). In the single factor analysis, no significant associations were observed between any of all the five polymorphisms and gastric cancer risk before and after adjusting for age, gender, pack-years, smoking and drinking status. We then determined the risk genotypes for each SNP based on its association with gastric cancer susceptibility. If a genotype of a SNP was associated with increase gastric cancer risk [odds ratio (OR)>1], the genotype was considered as a risk genotype, even if the association was not significant. When we combined the five polymorphisms, we observed that carriers of 3-4 risk genotypes had a significantly increased gastric cancer risk by 32%, when compared to carriers of 0-2 risk genotypes [OR=1.32, 95% confidence interval (CI)=1.04-1.68, *P*=0.021]. However, this association were weakened and became borderline significant (adjusted OR=1.29, 95% CI=0.99-1.69, *P=*0.062) after adjustment for age, gender, pack-years, smoking and drinking status.

**Table 1:** Logistic regression analysis of associations between *XPG* and gastric cancer risk

Genotypes	Cases (n=1142)	Controls (n=1173)	*P*^a^	Crude OR (95% CI)	*P*	Adjusted OR (95% CI) ^b^	*P*^b^
rs2094258							
CC	499 (43.70)	527 (44.93)		1.00		1.00	
CT	508 (44.48)	524 (44.67)		1.02 (0.86-1.22)	0.789	0.99 (0.82-1.21)	0.938
TT	135 (11.82)	122 (10.40)		1.17 (0.89-1.54)	0.265	1.17 (0.86-1.59)	0.329
Dominant	643 (56.30)	646 (55.07)	0.551	1.05 (0.89-1.24)	0.551	1.03 (0.85-1.24)	0.794
Additive model			0.534	1.06 (0.94-1.20)	0.338	1.05 (0.91-1.21)	0.488
Recessive	1007 (88.18)	1051 (89.60)	0.277	1.16 (0.89-1.50)	0.277	1.17 (0.87-1.57)	0.291
rs751402							
CC	426 (37.30)	433 (36.91)		1.00		1.00	
CT	555 (48.60)	551 (46.97)		1.02 (0.86-1.22)	0.796	1.09 (0.89-1.34)	0.397
TT	161 (14.10)	189 (16.11)		0.87 (0.68-1.11)	0.258	0.87 (0.65-1.15)	0.328
Dominant	716 (62.70)	740 (63.09)	0.846	0.98 (0.83-1.16)	0.846	1.03 (0.85-1.25)	0.740
Additive model			0.387	0.95 (0.84-1.07)	0.401	0.97 (0.84-1.10)	0.606
Recessive	981 (85.90)	984 (83.89)	0.176	0.86 (0.68-1.07)	0.177	0.83 (0.64-1.07)	0.150
rs2296147							
TT	725 (63.49)	746(63.60)		1.00		1.00	
CT	364 (31.87)	388 (33.08)		0.97 (0.81-1.15)	0.694	0.98 (0.80-1.20)	0.856
CC	53 (4.64)	39 (3.32)		1.40 (0.91-2.14)	0.123	1.28 (0.78-2.08)	0.329
Dominant	417 (36.51)	427 (36.40)	0.955	1.01 (0.85-1.19)	0.955	1.01 (0.83-1.22)	0.927
Additive model			0.249	1.05 (0.91-1.21)	0.544	1.04 (0.88-1.22)	0.672
Recessive	1089 (95.36)	1134 (96.68)	0.105	1.42 (0.93-2.16)	0.107	1.28 (0.79-2.09)	0.312
rs1047768							
TT	607 (53.15)	625 (53.28)		1.00		1.00	
TC	445 (38.97)	461 (39.30)		0.99 (0.84-1.18)	0.944	0.96 (0.79-1.17)	0.706
CC	90 (7.88)	87 (7.42)		1.07 (0.78-1.46)	0.695	1.10 (0.77-1.58)	0.591
Dominant	535 (46.85)	548 (46.72)	0.950	1.01 (0.85-1.18)	0.950	0.98 (0.82-1.19)	0.869
Additive model			0.913	1.02 (0.89-1.15)	0.822	1.01 (0.87-1.17)	0.891
Recessive	1052 (92.12)	1086 (92.58)	0.674	1.07 (0.79-1.45)	0.675	1.12 (0.79-1.59)	0.521
rs873601							
GG	311 (27.23)	323 (27.54)		1.00		1.00	
AG	557 (48.77)	598 (50.98)		0.97 (0.80-1.17)	0.738	0.97 (0.78-1.21)	0.796
AA	274 (23.99)	252 (21.48)		1.13 (0.90-1.42)	0.303	1.11 (0.85-1.44)	0.448
Dominant	831 (72.77)	850 (72.46)	0.870	1.02 (0.85-1.22)	0.870	1.01 (0.82-1.25)	0.909
Additive model			0.335	1.06 (0.94-1.19)	0.338	1.05 (0.92-1.20)	0.480
Recessive	868 (76.01)	921 (78.52)	0.150	1.15 (0.95-1.40)	0.150	1.13 (0.91-1.41)	0.286
Risk genotypes							
0	158 (13.84)	184 (15.69)	0.185	1.00		1.00	
1	613 (53.68)	638 (54.39)		1.12 (0.88-1.42)	0.358	1.16 (0.88-1.52)	0.295
2	194 (16.99)	208 (17.73)		1.09 (0.81-1.45)	0.575	1.13 (0.81-1.57)	0.471
3	176 (15.41)	142 (12.11)		**1.44 (1.06-1.96)**	**0.019**	**1.45 (1.02-2.05)**	**0.038**
4	1 (0.09)	1 (0.09)		1.17 (0.07-18.77)	0.915	2.61 (0.06-107.90)	0.614
0-2	965 (84.50)	1030 (87.81)		1.00		1.00	
3-4	177 (15.50)	143 (12.19)	0.021	**1.32 (1.04-1.68)**	**0.021**	1.29 (0.99-1.69)	0.062

CI, confidence interval; OR, odds ratio.

a Chi square test for genotype distributions between cases and controls.

b Adjusted for age, gender, pack-years, smoking and drinking status in logistic regress models.

### Stratification analysis

In the stratified analysis by age, gender, smoking status, pack-year, drinking status, tumor sites and TNM stage, we further evaluated the effects of all the five SNPs and provided the results for rs751402 C>T, rs873601 G>A polymorphisms. The effects of combined risk genotypes on gastric cancer risk were also shown. We failed to find any significant association with gastric cancer risk for any studied variants among subgroups (Table 2). When the risk genotypes were combined, the significant associations with 3-4 risk genotypes were observed in individuals older than 58 years (adjusted OR=1.90, 95% CI=1.06-3.41, *P*=0.030) and men (adjusted OR=1.50, 95% CI=1.07-2.11, *P*=0.019), when compared to 0-2 risk genotypes.

**Table 2:** Stratification analysis for associations between the three *XPG* variant genotypes and gastric cancer risk in Chinese population

Variables	rs751402 (cases/controls)		Adjusted OR (95% CI)	*P* ^a^	rs873601 (cases/controls)		Adjusted OR (95% CI)	*P* ^a^	Risk genotype (case/control)		Adjusted OR (95% CI)	*P* ^a^
	CC/CT	TT			GG/AG	AA			0-2	3-4		
Median age, yr												
≤58	508/850	90/166	0.89 (0.67-1.20)	0.454	448/796	150/220	1.19 (0.93-1.53)	0.163	511/888	87/128	1.14 (0.84-1.55)	0.407
>58	473/134	71/23	0.82 (0.49-1.36)	0.436	420/125	124/32	1.12 (0.72-1.75)	0.604	454/142	90/15	**1.90 (1.06-3.41)**	**0.030**
Gender												
Males	637/655	112/134	0.84 (0.61-1.15)	0.264	567/625	182/164	1.17 (0.89-1.55)	0.267	629/695	120/94	**1.50 (1.07-2.11)**	**0.019**
Females	344/329	49/55	0.83 (0.53-1.31)	0.431	301/296	92/88	1.07 (0.74-1.55)	0.708	336/335	57/49	0.98 (0.63-1.54)	0.940
Smoking status												
Never	633/559	102/103	0.77 (0.55-1.08)	0.131	555/521	180/141	1.27 (0.94-1.68)	0.123	616/582	119/80	1.28 (0.90-1.82)	0.167
Ever	348/425	59/86	0.89 (0.60-1.34)	0.590	313/400	94/111	0.98 (0.68-1.40)	0.896	349/448	58/63	1.24 (0.80-1.92)	0.330
Pack-year												
0	633/559	102/103	0.77 (0.55-1.08)	0.131	555/521	180/141	1.27 (0.94-1.68)	0.123	616/582	119/80	1.28 (0.90-1.82)	0.167
≤ 30	231/322	41/61	1.04 (0.63-1.71)	0.891	211/295	61/88	0.93 (0.60-1.44)	0.750	232/331	40/52	1.04 (0.62-1.75)	0.894
> 30	117/103	18/25	0.70 (0.35-1.41)	0.313	102/105	33/23	1.06 (0.56-2.03)	0.857	117/117	18/11	1.73 (0.73-4.09)	0.211
Drinking status												
Never	795/499	139/101	0.84 (0.62-1.13)	0.237	708/470	226/130	1.16 (0.89-1.50)	0.280	790/522	144/78	1.17 (0.85-1.61)	0.326
Ever	186/485	22/88	0.77 (0.44-1.35)	0.357	160/451	48/122	1.11 (0.71-1.74)	0.659	175/508	33/65	1.46 (0.85-2.50)	0.169
Tumor site												
Cardia	205/984	35/189	0.88 (0.57-1.36)	0.554	185/921	55/252	1.14 (0.78-1.66)	0.500	205/1030	35/143	1.39 (0.88-2.18)	0.156
Non-cardia	776/984	126/189	0.81 (0.62-1.07)	0.141	683/921	219/252	1.14 (0.90-1.43)	0.279	760/1030	142/143	1.31 (0.99-1.74)	0.059
Duke stage												
I/II	405/984	64/189	0.75 (0.53-1.06)	0.106	360/921	109/252	1.13 (0.85-1.51)	0.407	397/1030	72/143	1.31 (0.92-1.86)	0.130
III/IV	576/984	97/189	0.88 (0.65-1.18)	0.397	508/921	165/252	1.16 (0.90-1.48)	0.261	568/1030	105/143	1.33 (0.98-1.81)	0.066

CI, confidence interval; OR, odds ratio.

a Obtained in logistic regression models with adjustment for age, gender, pack-years, smoking and drinking status with omitting the corresponding stratification factor.

### Haplotype analysis

The frequency of inferred haplotypes of *XPG* gene based on observed genotypes and their association with the risk of gastric cancer were shown in Table 3. None of the haplotype was associated with gastric cancer risk significantly.

**Table 3:** The frequency of inferred haplotypes of *XPG* gene based on observed genotypes and their association with the risk of gastric cancer

Haplotypes ^a^	Cases (n=2284)	Controls (n=2346)	Crude OR (95% CI)	*P*	Adjusted OR ^b^ (95% CI)	*P* ^b^
CTTTG	747 (32.71)	802 (34.19)	1.00		1.00	
CTTTA	123 (5.39)	122 (5.20)	1.08 (0.83-1.42)	0.565	1.06 (0.78-1.44)	0.694
CTTCG	4 (0.18)	5 (0.21)	0.86 (0.23-3.21)	0.821	0.64 (0.15-2.75)	0.547
CTTCA	1 (0.04)	0	/	0.978	/	0.981
CTCTA	1 (0.04)	0	/	0.978	/	0.980
CCTTG	140 (6.13)	150 (6.39)	1.00 (0.78-1.29)	0.987	0.92 (0.70-1.23)	0.584
CCTTA	67 (2.93)	80 (3.41)	0.90 (0.64-1.26)	0.540	0.80 (0.54-1.18)	0.253
CCTCG	31 (1.36)	48 (2.05)	0.69 (0.44-1.10)	0.121	0.82 (0.49-1.40)	0.468
CCTCA	73 (3.20)	66 (2.81)	1.19 (0.84-1.68)	0.332	1.17 (0.79-1.72)	0.443
CCCTG	5 (0.22)	8 (0.34)	0.67 (0.22-2.06)	0.486	0.76 (0.21-2.76)	0.679
CCCTA	2 (0.09)	2 (0.09)	1.07 (0.15-7.64)	0.943	1.58 (0.19-13.31)	0.674
CCCCG	194 (8.49)	177 (7.54)	1.18 (0.94-1.48)	0.160	1.14 (0.88-1.47)	0.330
CCCCA	118 (5.17)	118 (5.03)	1.07 (0.82-1.41)	0.611	1.16 (0.85-1.58)	0.358
TTTTG	1 (0.04)	0	/	0.978	/	0.976
TCTTG	40 (1.75)	43 (1.83)	1.00 (0.64-1.55)	0.996	1.02 (0.62-1.68)	0.925
TCTTA	528 (23.12)	498 (21.23)	1.14 (0.97-1.33)	0.108	1.13 (0.95-1.36)	0.169
TCTCG	6 (0.26)	2 (0.09)	3.22 (0.65-16.01)	0.153	2.10 (0.40-11.05)	0.383
TCTCA	53 (2.32)	64 (2.73)	0.89 (0.61-1.30)	0.542	0.87 (0.57-1.34)	0.538
TCCTA	5 (0.22)	6 (0.26)	0.90 (0.27-2.94)	0.855	1.07 (0.30-3.83)	0.918
TCCCG	11 (0.48)	9 (0.38)	1.31 (0.54-3.18)	0.548	1.41 (0.51-3.91)	0.504
TCCCA	134 (5.87)	146 (6.22)	0.99 (0.76-1.27)	0.910	0.89 (0.66-1.18)	0.411

a The haplotypes order were rs2094258, rs751402, rs2296147, rs1047768, and rs873601.

b Obtained in logistic regression models with adjustment for age, gender, pack‐years, smoking and drinking status.

## DISCUSSION

In the present study, we investigated the impact of five potentially functional *XPG* SNPs on gastric cancer risk in a Chinese Han population from South China. Our analysis indicated that none of these SNPs could individually influence the gastric cancer susceptibility. However, the individuals carrying 3-4 risk genotypes had a significantly increased gastric cancer risk, especially among those older than 58 years and men. To the best of our knowledge, this is the largest study to investigate the association of these five *XPG* polymorphisms with the gastric cancer risk by far.

XPG is an indispensable component of the NER pathway, which is responsible for the cleavage of DNA on the 3′ side of lesion and also recruit PCNA to the damage sites for the subsequent gap-filling DNA synthesis in mammals [31]. It is reported that XPG also participates in other cellular processes, such as transcription-coupled DNA repair and RNA polymerase II transcription [32, 33].

Recently, several studies have been carried out to explore the role of *XPG* polymorphisms in gastric cancer susceptibility; however, inconsistent results have been reported. We previously evaluated the association between *XPG* (rs2094258 C>T, rs2296147 T>C and rs873601 G>A) and gastric cancer risk in an Eastern Chinese population with 1125 cases and 1196 controls. We found that the rs873601 G>A polymorphism (located in the 3′ UTR) was significantly associated with an increased gastric cancer risk [22]. We also demonstrated that rs873601 A allele was significantly associated with reduced mRNA expression level of *XPG* gene. These three polymorphisms were also genotyped in 337 gastric cancer cases and 347 controls by Yang and coworkers [24]. Intriguingly, they found that the rs2296147 T>C polymorphism was associated with a decreased gastric cancer risk, while the rs2094258 C>T polymorphism was associated with an increased gastric cancer risk [24]. In a study by Duan et al. [21], composed of 400 gastric cancer cases and 400 healthy controls, both rs751402 C>T and rs2296147 T>C polymorphisms were shown to significantly increase gastric cancer risk. Recently, Chen et al. [23] explored the association of rs2094258 C>T, rs751402 C>T, rs2296147 T>C and rs873601 G>A polymorphisms with gastric cancer susceptibility in 692 cases and 771 healthy controls. However, only *XPG* rs873601 G>A polymorphism appeared to be associated with the risk of gastric cancer. This controversy regarding the association might be partly due to ethnic and demographic differences, or insufficient statistical power caused by small sample size.

With this in mind, we conducted the current study with 1142 cases and 1173 controls. We found no significant association between variant genotypes of *XPG* polymorphisms and gastric cancer risk. However, the individuals carrying 3-4 risk genotypes were at significantly increased gastric cancer risk, especially for individuals older than 58 years and men. Overall, the negative results might be partially ascribed to the mild effect of each variant. In addition, the moderate sample size in this study might not be large enough to detect relatively weak association. Besides, complex interactions between environmental and genetic factors should be taken into account while measuring the true associations of *XPG* gene polymorphisms with gastric cancer.

Despite that this is the largest study to extensively analyze the association of five potentially functional *XPG* polymorphisms with gastric cancer in a Southern Chinese population, there still exists some limitations. First, frequency matching between cases and controls in this research were only performed on gender, but not on age, smoking and drinking status. We used multivariate logistic regression analysis to minimize the impact of these confounding factors, to some extent. Second, gastric cancer is a heterogeneous disease which might be influenced by other related factors such as *H. pylori* infection, diet, occupational exposure, and environmental factors. Since such information on participants was missing, the results should be explained with caution. Third, due to the hospital-based case-control design, our study was inevitably suffered from the selection bias. Moreover, the conclusions drawn from subjects residing in South China may not well represent other Chinese populations in the different regions. Fourth, only five potentially functional SNPs were included in this study. As a result, SNPs from the coding and the intron regions that may also be related to gastric cancer risk could be omitted. Finally, we only investigated the association between *XPG* gene polymorphisms and gastric cancer risk. Genetic variations in other genes (e.g. *KDM5A*, *DNAH7* [34], *PLCE1* [35], *PSCA* [36, 37], *PRKAA1* [38], *MUC1* [39]) reported to be specifically associated with gastric cancer initiation and progression were not investigated in the current study.

In conclusion, we found that none of the *XPG* rs2094258 C>T, rs751402 C>T, rs2296147 T>C, rs1047768 T>C and rs873601 G>A polymorphisms was associated with gastric cancer susceptibility. However, cumulative effects of risk genotypes (3-4) on the risk of gastric cancer were observed. Further well-designed, prospective studies with large-scale multicenter investigations involving different ethnicities are required to verify our conclusions.

## MATERIALS AND METHODS

### Study subjects

The study protocol was approved by the institutional review board of Sun Yat-sen University Cancer Center. All participants of this study signed individual informed consent. This study consisted of 1142 patients and 1173 healthy controls as we describe previously [40]. All subjects were unrelated ethnic Han Chinese population from Southern China, mainly from Guangdong, Guangxi, and Hainan province. In general, the response rate of cases and controls was more than 85%.

### SNP selection and genotyping

Five potentially functional SNPs in the *XPG* gene were selected for this study as we described previously [28, 29]. Briefly, we searched the potentially functional candidate SNPs located in the 5′- flanking region, exon, 5′ UTR, and 3′ UTR, which might affect transcription activity and the microRNA binding site activity. As predicted by SNPinfo software (http://snpinfo.niehs.nih.gov/snpinfo/snpfunc.htm), five SNPs (rs2094258 C>T, rs751402 C>T, rs2296147 T>C, rs1047768 T>C and rs873601 G>A) were potentially functional (Supplemental Table 2). All these SNPs have a minor allele frequency no less than 5% for Chinese Han subjects. There is no significant linkage disequilibrium (LD) (R^2^<0.8) among these SNPs. DNA samples were genotyped by the Taqman real-time PCR method as we described previously [22, 41].

### Statistical analysis

Goodness-of-fit *χ^2^* test was used to check whether genotype frequency distribution of each polymorphism in controls were in accordance with HWE. We compared the differences in demographic variables as well as genotype frequencies between cases and controls by using the two-sided *χ^2^* test. ORs and 95% CIs were used to estimate the effect of SNPs and haplotypes on gastric cancer risk. Adjusted ORs were calculated by unconditional multivariate logistic regression analysis, with adjustment for age, gender, pack-years, smoking and drinking status. We determined the risk genotypes for each SNP based on its association with gastric cancer susceptibility. If a genotype of a SNP was shown to increase gastric cancer risk (OR>1), the genotype was regarded as a risk genotype. For example, as to the rs2094258 C>T polymorphism, ORs of 1.02 (heterozygous model) and 1.17 (homozygous model) indicated that the T allele carriers (CT/TT) may have an increased risk when compared to those with CC genotypes (Table 1). Thus, the CC wild-type genotype carriers was define as 0, while the CT or TT genotype carriers was defined as 1. We then divided subjects into two groups based the number of risk genotypes. Carriers of 3-4 risk genotypes represented those carrying 3-4 risk genotypes of the five SNPs, while 0-2 risk genotypes represented those carrying 0-2 risk genotypes. All statistical analyses were performed using SAS software (version 9.1; SAS Institute, Cary, NC). A *P* value of <0.05 was considered as statistically significant.

## SUPPLEMENTARY MATERIAL TABLES


