# Supplementary material for: Association between genetic variants in the XPG gene and gastric cancer risk in a Southern Chinese population
Source: Aging (Albany NY). 2016 Dec 6;8(12):3311–9. doi: 10.18632/aging.101119 (PMC5270670; doi:10.18632/aging.101119)
Supplement: Supplementary file 1 [file aging-08-3311-s001.pdf]

SUPPLEMENTARY MATERIAL

Supplemental Table S1. Frequency distribution of selected characteristics in gastric cancer cases and controls.

Variables	Cases (n=1142)		Controls (n=1173)		P^a
	No	%	No.	%	
Gender					0.393
Males	749	65.59	789	67.26	
Females	393	34.41	384	32.74	
Age range, yr	15-86		16-80		<0.0001
Mean ± SD	56.25 ± 12.49		45.19 ± 11.56		
≤ 50	334	29.25	789	67.26	
51-60	362	31.70	285	24.30	
61-70	312	27.32	73	6.22	
>70	134	11.73	26	2.22	
Smoking status					<0.0001
Never	735	64.36	662	56.44	
Ever	407	35.64	511	43.56	
Pack-years					<0.0001
0	735	64.36	662	56.44	
≤ 30 (mean)	272	23.82	383	32.65	
> 30 (mean)	135	11.82	128	10.91	
Drinking status					<0.0001
No	934	81.79	600	51.15	
Yes	208	18.21	573	48.85	
Sites					
Cardia	240	21.02	/	/	
Non-cardia	902	78.98	/	/	
TNM Stage					
I	140	12.26	/	/	
II	329	28.81	/	/	
III	456	39.93	/	/	
IV	217	19.00	/	/	

[a] Two-sided χ^2 test for distributions between gastric cancer cases and controls.

Supplemental Table S2. The potential functions of the five SNPs selected in *XPG* gene as predicted by SNPinfo (http://snpinfo.niehs.nih.gov/snpinfo/snpfunc.htm) software.

rs	Chr	Allele	Position [a]	Location	TFBS	Splicing (ESE or ESS)	miRNA (miRanda)	miRNA (Sanger)	Allele	Asian	CHB
rs2094258	13	C/T	102294760	5' near gene	Y	--	--	--	C	0.627	0.661
rs751402	13	C/T	102296199	5' UTR	Y	Y	--	--	C	0.591	0.619
rs2296147	13	T/C	102296376	5' UTR	Y	--	--	--	T	0.838	0.768
rs1047768	13	T/C	102302518	exon	--	Y	--	--	T	0.778	0.720
rs873601	13	G/A	102326338	3' UTR	--	Y	Y	Y	G	0.517	0.464

[a] The position is based on HapMap Data Rel 27 Phase II+III, Feb09, on NCBI B36 assembly, dbSNP b126

SNP, single nucleotide polymorphism; *XPG*, xeroderma pigmentosum group G; TFBS, transcription factor binding site; ESE, exon splicing enhancer; ESS, exon splicing silencer; CHB, Han Chinese in Beijing, China; UTR, untranslated region.
